# Enterotoxigenic *Clostridium perfringens*: Detection and Identification

**DOI:** 10.1264/jsme2.ME12002

**Published:** 2012-04-14

**Authors:** Kazuaki Miyamoto, Jihong Li, Bruce A. McClane

**Affiliations:** 1 Department of Microbiology, Wakayama Medical University School of Medicine, 811–1 Kimiidera, Wakayama 641–0012, Japan; 2 Department of Microbiology and Molecular Genetics, University of Pittsburgh School of Medicine, Pittsburgh, Pennsylvania 15261, USA

**Keywords:** molecular assays, *cpe*-genotyping assay, MLST

## Abstract

Recent advances in understanding the genetics of enterotoxigenic *Clostridium perfringens*, including whole genome sequencing of a chromosomal *cpe* strain and sequencing of several *cpe*-carrying large plasmids, have led to the development of molecular approaches to more precisely investigate isolates involved in human gastrointestinal diseases and isolates present in the environment. Sequence-based PCR genotyping of the *cpe* locus (*cpe* genotyping PCR assays) has provided new information about *cpe*-positive type A *C. perfringens* including: 1) Foodborne *C. perfringens* outbreaks can be caused not only by chromosomal *cpe* type A strains with extremely heat-resistant spores, but also less commonly by less heat-resistant spore-forming plasmid *cpe* type A strains; 2) Both chromosomal *cpe* and plasmid *cpe C. perfringens* type A strains can be found in retail foods, healthy human feces and the environment, such as in sewage; 3) Most environmental *cpe*-positive *C. perfringens* type A strains carry their *cpe* gene on plasmids. Moreover, recent studies indicated that the *cpe* loci of type C, D, and E strains differ from the *cpe* loci of type A strains and from the *cpe* loci of each other, indicating that the *cpe* loci of *C. perfringens* have remarkable diversity. Multi-locus sequence typing (MLST) indicated that the chromosomal *cpe* strains responsible for most food poisoning cases have distinct genetic characteristics that provide unique biological properties, such as the formation of highly heat-resistant spores. These and future advances should help elucidate the epidemiology of enterotoxigenic *C. perfringens* and also contribute to the prevention of *C. perfringens* food poisoning outbreaks and other CPE-associated human diseases.

## Introduction

*Clostridium perfringens* is a Gram-positive, rod-shaped, spore-forming, anaerobic bacterium that causes a broad spectrum of human and veterinary diseases (35, 36). The virulence of *C. perfringens* largely results from its prolific toxin-producing ability (36). Based on the production of four major toxins (alpha, beta, epsilon, and iota), this organism is commonly classified into one of five types (A to E) (35). Some *C. perfringens* strains produce another important toxin named *Clostridium perfringens* enterotoxin (CPE), which is responsible for several human gastrointestinal (GI) diseases, including *C. perfringens* type A food poisoning and many cases of antibiotic-associated diarrhea (AAD), sporadic diarrhea (SD), and nosocomial diarrheal disease (1, 2, 23, 25, 52, 56). Therefore, detection of CPE produced by *C. perfringens* in feces specimens of ill individuals is a criterion for clinical diagnosis.

CPE production, which is responsible for the diarrhea symptoms of diseases caused by *cpe*-positive type A strains, is sporulation-associated. Intact *cpe* genes can also be found in some type C, D and E strains. CPE expression is also sporulation-associated in those type C and D strains and, probably, also in those type E isolates, based upon sequence data indicating the presence of sigE- and sigK-dependent promoters upstream of the *cpe* gene in those type E strains (10, 16, 18, 32, 43, 50, 59).

Despite the medical importance of enterotoxigenic *C. perfringens*, the ecology of these bacteria remains poorly understood. In part this is because, while *C. perfringens* has widespread distribution in the environment, only a small fraction (~1 to 5%) of the global *C. perfringens* population carries the enterotoxin (*cpe*) gene (8, 14, 24, 29, 34, 38, 44, 55, 57). However, enterotoxigenic *C. perfringens* are a suitable target bacterium for microbial source tracking (MST) for identifying contamination processes (21). Recently, the accumulation of genetic information about chromosomal and plasmid *cpe* type A strains has facilitated the development of molecular methods using MST tools for detecting and identifying enterotoxigenic *C. perfringens* (41, 42, 45, 48, 58). These molecular methods to detect the *cpe* gene and to identify the *cpe* locus represent a useful alternative approach for MST (41, 58). Using recently developed molecular assays, several new findings about enterotoxigenic *C. perfringens* ecology have been reported (29, 44); therefore, new strategies for preventing human and animal GI diseases caused by enterotoxigenic *C. perfringens* may be developed in the near future.

## Molecular assays for detecting the *cpe* gene

*C. perfringens* type A food poisoning usually develops after the ingestion of foods contaminated with large numbers (>10^6^ bacteria g^−1^) of CPE-positive vegetative cells (36). Those bacteria then sporulate in the intestines and produce CPE. The stool from diseased persons typically contains large numbers (>10^6^ bacteria g^−1^) of CPE-positive *C. perfringens* spores (36). To prove *C. perfringens* as the etiologic agent of an outbreak, serotyping or molecular genotyping assays, such as pulsed-field gel electrophoresis (PFGE) have been developed (23, 33).

However, in some outbreaks, enterotoxigenic *C. perfringens* can only be isolated from feces of sick individuals and not from any food source, and only low numbers of viable bacteria remain in those feces (this is a particular problem if fecal samples are not collected soon after the onset of diarrhea). To identify the contaminated food in these cases, molecular methods such as conventional PCR, nested PCR, real-time PCR, and other recently developed assays, such as loop-mediated isothermal amplification (LAMP assay) can be useful tools (21). In these assays, ~10^3^ *cpe*-positive bacterial cells are necessary for detection, while these assays can detect 0.1 to 10 pg of purified bacterial DNA; however, combined with enrichment culture, these assays can detect less than three viable *cpe*-positive *C. perfringens* strains (21); therefore, these assays are also helpful to identify how and when enterotoxigenic *C. perfringens* isolates enter the food supply. The results of future surveys using molecular assays will be useful to fully understand and prevent *C. perfringens* type A food poisoning outbreaks.

These assays have been applied to isolates from food poisoning outbreaks, diarrheic patients, and other sources. It is often easier to detect the *cpe* gene with a molecular assay than to detect *in vitro* CPE production by strains, because the production of CPE is sporulation-associated, *i.e.*, demonstrating *in vitro* CPE production requires the *in vitro* sporulation of isolates (3, 18, 59). Unfortunately, sporulation is often difficult to achieve *in vitro* using sporulation media such as Duncan-Strong medium (36).

Despite the advantages of molecular methods for detecting the presence of the *cpe* gene when screening many samples, an epidemiological protocol for detecting *cpe*-positive strains has not been fully established. The reasons are: 1) The presence of *cpe*-positive *C. perfringens* in non-outbreak foods and the environment is often at a low frequency of 1 to 5% or less and it is expensive and time consuming to identify *cpe*-positive *C. perfringens* from large numbers of isolates; 2) In non-outbreak retail foods, the number of contaminated *C. perfringens* is usually very low, less than 3 MPN/gram in many samples (38, 57); and 3) To detect *cpe*-positive *C. perfringens* with molecular assays, a large number of bacteria, (approximately 10^3^ g^−1^ sample) are necessary to prepare template DNA using commercial DNA purification kits (21). Because of these difficulties, studies using molecular methods for the detection of *cpe*-positive *C. perfringens* in food samples have rarely been published (38, 39).

To overcome these issues, an additional enrichment culture step is usually used before the preparation of DNA template for PCR analysis (21, 38, 46, 57). Adding an enrichment culture step allowed the isolation and detection of enterotoxigenic *C. perfringens* from retail uncooked food samples (21, 38, 57). In a recent study using molecular methods for detecting *cpe*-positive *C. perfringens*, the addition of an enrichment culture step markedly improved the detection efficiency of the *cpe* gene in retail raw meat samples (21). Moreover, an enrichment culture can reduce known (collagen molecule) and unknown inhibitors present in food samples (21).

Although molecular methods can detect non-viable *cpe-*positive *C. perfringens* in tested samples, significant numbers of these bacteria are needed to obtain a PCR-positive sample, *i.e.*, *cpe*-positive *C. perfringens* should be propagated in samples before testing. Even using an enrichment culture step, not all *cpe*-positive *C. perfringens* might be detected in all samples if many more *cpe*-negative *C. perfringens* are present in the sample.

Collectively, many issues remain for detecting *cpe-*positive *C. perfringens* in foods and the environment for epidemiological purposes, although repressing food contamination by *cpe*-positive *C. perfringens* is important to reduce the occurrence of *C. perfringens* food poisoning outbreaks.

## Genetic diversity of *cpe* loci amongst type A, C, D and E *cpe*-carrying *C. perfringens*

In type A isolates, the *cpe* gene can reside on the *C. perfringens* chromosome or on large plasmids (12). Some type C, D, and E, isolates also carry functional *cpe* genes on large plasmids (32, 43). In most or all CPE-positive type A, C, and D isolates, the *cpe* gene encodes a 957 bp ORF whose sequence is identical; however, some type E isolates encode a variant of the functional *cpe* gene, while other type E isolates carry a silent *cpe* gene (10, 12, 13, 30, 43).

Early studies indicated that classical type A food poisoning *C. perfringens* isolates carry a chromosomal *cpe* gene (11, 12). It was also reported that CPE-positive *C. perfringens* type A isolates causing non-foodborne human diarrhea disease such as AAD and SD carry their *cpe* gene on a large plasmid (5, 6, 11, 12).

**Fig. 1:**
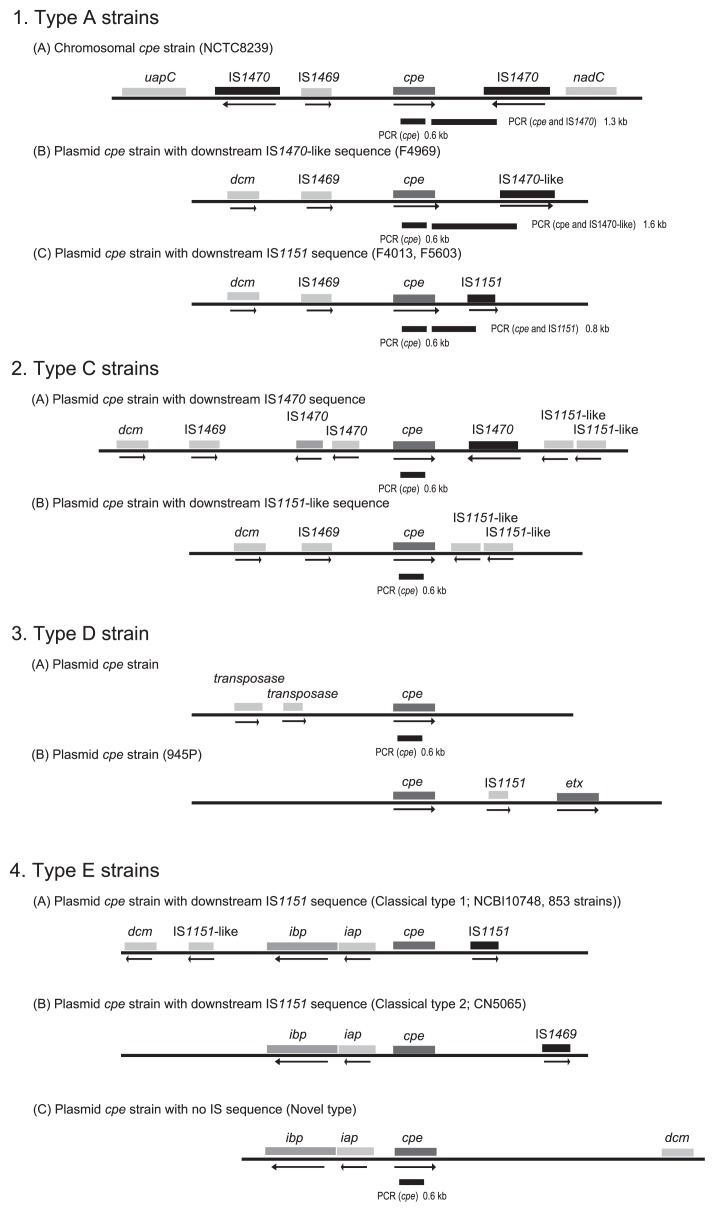
The organization of *cpe* loci in *cpe*-positive type A, C, D, and E *C. perfringens*. In type A strains, comparison of the genetic organization of chromosomal *cpe* locus (A), and plasmid *cpe* loci (B, C) revealed three downstream sequences. The bar below the open reading frames depicts the *cpe*-IS*1470 cpe* genotyping PCR assay product from chromosomal *cpe* strains, from *cpe*-IS*1470*-like plasmid strains, and from *cpe*-IS*1151* plasmid strains. In type C strains, two types of genetic organization of plasmid *cpe* loci have been found, *i.e.*, (A) *cpe* locus with downstream IS*1470* and two IS*1151*-like sequences and (B) the *cpe* locus with two IS*1151*-like sequences. In type D strains, the genetic organization of the *cpe* locus lacks any apparent IS sequence. In type E strains, the genetic organization of (A) iota toxin locus with the disrupted *cpe* gene and IS*1151*, (B) iota toxin locus with the disrupted *cpe* gene and IS*1469*, and (C) iota toxin locus with functional *cpe* gene and no apparent IS element. The bar below the open reading frames of the *cpe* gene depicts the *cpe* genotyping PCR assay product by internal primers of the *cpe* gene.

Comparing the organization of the chromosomal *cpe* locus versus plasmid *cpe* loci in type A isolates revealed an identical ~3 kb region, which contains an upstream IS*1469* element, the *cpe* gene, and downstream sequences, present in both the plasmid and chromosomal *cpe* loci (Fig. 1) (7, 40). Beyond this conserved region immediately surrounding the *cpe* gene, substantial differences were identified between these *cpe* loci amongst type A isolates. When chromosomally-located, the *cpe* gene appears to be associated with mobile genetic elements, *i.e.*, the chromosomal *cpe* gene is present on a putative 6.3 kb transposon, named Tn*5565* (7). This putative transposon is flanked by upstream and downstream copies of IS*1470* and is apparently inserted between the purine permease (*uapC*) and quinolinate phosphoribosyltransferase (*nadC*) genes on the chromosome (Fig. 1) (7).

The type A plasmid *cpe* locus lacks the IS*1470* element that is present upstream of the chromosomal *cpe* gene, instead carrying an upstream cytosine methyltransferase gene (*dcm*) (Fig. 1) (40, 42). Additionally, the IS*1470* element located downstream of the chromosomal *cpe* locus has been replaced in type A plasmid loci by either a defective IS*1470*-like element, or by an IS*1151* element (40, 42). Moreover, a study determining complete sequence and diversity analysis determined that the two kinds of plasmid *cpe* genotypes (downstream IS*1151* or downstream IS*1470*-like sequence) share a conserved region, including a replication region and a plasmid conjugative transfer region, but have different variable regions that can encode bacteriocins, metabolic genes or toxin genes, such as the *cpb2* gene (42).

Some strains of type C, D, and E *C. perfringens* also carry the *cpe* gene (4, 32, 43). The organization of the *cpe* loci of type C, and D isolates is different from that of type A plasmid *cpe* strains, while the *cpe* ORF sequence is identical amongst type A, C, and D isolates (32). Flanking sequences of the *cpe* gene in type C strains can be divided into two groups with one group carrying an upstream IS*1470* sequence and downstream IS*1470* and IS*1151*-like sequences, and the other group sharing a resemblance to the plasmid-borne *cpe* locus of pCPF5603 carried by type A isolate F5603 (Fig. 1). Both types of the type C *cpe* locus are located downstream of the cytosine methyltransferase gene (*dcm*), which is almost always located upstream of the *cpe* gene in type A strains (25, 32, 40). Overall, the *cpe* gene has been localized near *dcm* in those *cpe* loci where the *cpe* gene is flanked by various combinations of IS*1469*, IS*1470*, IS*1470*-like, IS*1151* or IS*1151*-like sequences in type A and C *C. perfringens* isolates. Investigated type D strains carry a unique *cpe* locus, which is different from that in any other characterized *cpe*-positive *C. perfringens*. In these type D isolates, upstream of the *cpe* gene, there are two copies of the putative transposase gene in Tn*154*; however, no IS element is found downstream of the *cpe* gene (Fig. 1) (32).

Amongst type E isolates, two groups of *cpe* loci have been identified (4, 43). In classical type E isolates, a silent *cpe* sequence associated with IS*1151* and IS*1469* is located on the same plasmid adjacent to the iota toxin genes (Fig. 1) (4, 47); however, in some recently-identified type E isolates, an intact *cpe* gene is next to the iota genes (Fig. 1) (43). Interestingly, the newly identified *cpe* gene in those the novel type E isolates has several nucleotide differences from the classical *cpe* gene of type A, C, and D isolates (32, 43). The iota toxin genes in these novel type E isolates also exhibit nucleotide differences from the classical iota toxin genes (43); therefore, this newly identified type E isolate was initially classified as type A by the current PCR-based toxin genotyping assay as the *iap* primer in this assay could not amplify the variant *iap* gene present in these type E isolates (17, 37, 51). Moreover, complete sequencing of this toxin plasmid showed that no intact transposase gene and a disrupted *dcm* gene are present on this putative conjugatively transferable toxin plasmid (43). Collectively, these studies have revealed greater diversity in the *cpe* gene and the *cpe* loci in type C, D, and E isolates than those in type A strains.

## Usefulness of cpe-genotyping assays

Using the sequence information generated for the *cpe* locus on the chromosome and plasmids in type A isolates, simple and rapid PCR *cpe* genotyping assays were developed (41, 58). Because these PCR assays can distinguish between the *cpe* locus on the chromosome and the two well characterized *cpe* loci present on the plasmids in type A isolates, these *cpe* genotyping assays are a useful diagnostic and epidemiological tools for investigating CPE-associated GI disease cases, including food poisoning, AAD, and SD caused by *cpe-*positive type A strains.

Applying these PCR based *cpe*-genotype assays to chromosomal or plasmid *cpe*-positive type A isolates from various sources, several new insights have been reported (34). First, ~30% of *C. perfringens* food poisoning outbreaks in Japan and Europe appear to be caused by type A plasmid *cpe* strains (19, 25, 53), with the remainder of *C. perfringens* food poisoning outbreaks caused by chromosomal *cpe* strains. Before sequencing analysis and development of sequence-based *cpe*-genotyping PCR assays, it had been thought that all food poisoning isolates carry *cpe* on their chromosome, while isolates from AAD and SD cases bear *cpe* on the plasmids (11, 12). This conclusion was based upon strains isolated from a limited number of food poisoning outbreaks that were investigated using RFLP Southern blotting analysis, an approach that can only be performed in research laboratories with a limited number of isolates. Second, the *cpe* genotyping assays, which can be easily performed with large numbers of isolates by clinical labs, identified chromosomal *cpe*-strains in ~1.4% of retail meats in USA (57). In contrast, ~4% of retail meat products in Japan were contaminated with type A plasmid *cpe*-positive strains, as determined using a PCR assay that can distinguish chromosomal or plasmid *cpe* strains (38). Also, three meat plasmid-*cpe* isolates were identified as being the IS*1470*-like *cpe* genotype and these isolates also formed relatively heat-labile spores, *i.e.*, D_100_ value of spores was less than 3 min in an isolate from sporadic diarrhea (38). These results indicated that both chromosomal *cpe* and plasmid *cpe* strains can contaminate food and potentially induce food poisoning, although the relative importance of these *cpe* genotypes for causing food poisoning may vary, perhaps due to cultural differences in food preparation. Third, chromosomal *cpe-*positive strains are rarely isolated from feces of healthy humans, although those feces do contain type A plasmid *cpe* strains (8, 19). Therefore, feces of healthy humans might be a reservoir for plasmid *cpe*-positive isolates and, perhaps less commonly, for chromosomal *cpe*-positive isolates. Because of their rarity, chromosomal *cpe* isolates might be found only transiently in healthy human feces. Fourth, at least two unusual variants of the *cpe* locus have been found amongst type A isolates recovered from foods, human feces and the environment, including sewage (19, 29, 34, 44). At least, some of these isolates with unusual *cpe* loci, obtained from feces of healthy humans, can produce CPE (19). These findings suggest that there might be several other *cpe* genotypes in type A isolates but the clinical significance of the variant *cpe* loci-carrying isolates has not yet been investigated; however, many of these *cpe* isolates that are untypeable by current genotype assays originated from the environment, such as sewage, and these isolates are rarely found in human feces and foods. These findings suggest a need for further investigation of how and when foods become contaminated with *cpe*-positive isolates. Use of molecular methods, including the *cpe* detection PCR method and/or *cpe*-genotyping PCR assay combined with prior enrichment culture, should facilitate these epidemiological studies (21, 38, 57).

Type C *cpe*-positive strains have been isolated from feces of patients suffering from enteritis necroticans (Pigbel) (26); however, the involvement, if any, of CPE in this disease is not fully understood because of the rarity of Pigbel and the lack of an established clinical diagnosis protocol. The involvement of type D *cpe*-positive strains in disease has also not yet been established (35). Use of recently developed *cpe-*genotyping assays should make it easier to distinguish these *cpe*-positive type C, D and/or E strains from type A *cpe-*positive isolates at the clinical stage, which might help the diagnosis and the investigation of *cpe*-positive type C, D, and E strains.

## Multi-locus sequence typing (MLST) of *C. perfringens*

To epidemiologically link isolates obtained from patients with isolates found in suspected food vehicles, classical serotype assays have been used; however, many *cpe*-positive food poisoning strains cannot be serotyped using existing antisera. As an alternative, pulsed-field gel electrophoresis (PFGE) has more recently been applied for the epidemiological study of isolates from *C. perfringens* food poisoning or from nosocomial outbreaks (23, 33). The PFGE method has advantages over serotyping for demonstrating a link between various isolates associated with an outbreak (23, 33). These advantages include high reproducibility, high typeability with substantial discrimination ability (more than 30 types are distinguishable), and applicability to many kinds of bacterial species.

While the PFGE approach can demonstrate a clonal lineage of outbreak strains, it does not reflect the properties based on gene sequence diversity; as a result, data interpretation might be subjective in some cases. In addition, in some *C. perfringens* strains, bacterial DNA is rapidly degraded partly by internal DNase of the bacterial cell; as a result, DNA fingerprinting shows smearing (22). On the other hand, sequence-based molecular analysis, an approach known as multi-locus sequence typing (MLST), makes it possible to investigate more precise relationships among isolates with respect to disease presentation and/or host preference; the major origin and route of pathogenic strain spread (15, 20, 48). Moreover, MLST is a very good candidate for bacterial genotyping because MLST generates unambiguous nucleotide sequence data and does not have the potential for subjectivity in data interpretation. In addition, this technique has high reproducibility, high typeability and applicability to many bacterial species, similar to PFGE (20, 54); therefore, MLST has been applied to a number of bacterial pathogens, with the subsequent creation of databases, to which new MLST data can be added as it is generated (http://www.mlst.net) (20, 54).

**Fig. 2:**
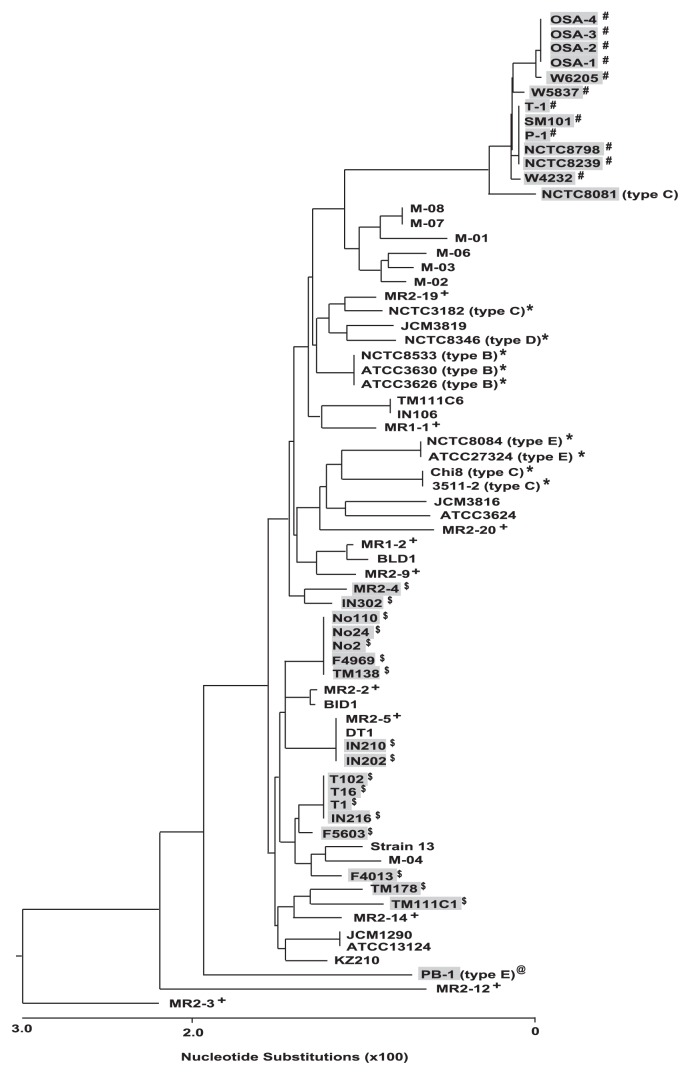
Phylogenetic relationships among *cpe*-positive and *cpe-*negative strains. The phylogenetic tree was constructed by Clustal W analysis based on the concatenated nucleotide sequence of 8 house-keeping genes. # indicates type A chromosomal *cpe* isolates from foods and food poisoning outbreaks. $ indicates type A plasmid *cpe* isolates from foods, sporadic diarrhea patients, food-borne outbreak, nosocomial outbreaks, and healthy individual. Isolates with a gray background are *cpe*-positive. * indicates type B to E animal diseases isolates. @ indicates novel type E isolate in retail meat sample. + indicates *cpe-*negative healthy human feces isolates.

The genes used in MLST analysis for *cpe*-positive *C. perfringens* isolates included genes for toxin genes (*plc*, *colA*), stress response (*groEL*, *sod*), putative metabolic genes (*pgk*, *nadA*), genes in DNA replication (*gyrB*) and genes for a sigma factor involved in sporulation (*sigK*), which is essential for *cpe* expression (15). These MLST studies indicated that human food poisoning isolates carrying the *cpe* gene on the chromosome are clearly related among strains isolated from several food poisoning outbreaks in Europe, United States, and Japan (Fig. 2) (15, 48). Plasmid-*cpe* isolates from human gastrointestinal diseases such as food-borne diarrhea and nosocomial diarrhea, were also clustered in the MLST assay, indicating that there also appeared to be a genetic relationship among plasmid-*cpe* isolates in feces of sickened individuals; however, *cpe*-negative isolates from healthy human feces exhibited considerable genetic diversity in the same MLST analysis (Fig. 2) (15).

By definition, toxin types B to E express one or more of beta, epsilon and iota toxins; as a result, these isolates are highly associated with specific diseases and animal hosts (9, 20). MLST approaches can identify host-species relationships with respect to the animal origin of isolates, even for isolates that are not clonal by PFGE profile (20, 48). Consistent with these findings, MLST indicated that type B to E strains from animal diseases was clustered with some diversity, while novel type E isolates from retail meat have a different genetic background from animal isolates (Fig. 2) (15, 43); however, there is no evidence that these type E meat isolates can cause food poisoning.

For *C. perfringens* isolates from various origins, the superoxide dismutase (*sod*) gene has been identified as a useful and sensitive PCR target gene for distinguishing chromosomal *cpe* strains from type A plasmid *cpe*-positive and other *cpe*-negative strains. This is because only a single locus sequence of the *sod* gene has been identified for chromosomal *cpe* strains (38).

## Other common properties of cpe-positive *C. perfringens*

Type A *C. perfringens* strains carrying the *cpe* gene on the chromosome, whether as vegetative cells or spores, usually possess much higher resistance properties against heat, cold, pH, and nitrites than type A strains carrying *cpe* on a plasmid (6, 27, 28, 31, 49). In addition, chromosomal *cpe* strains typically grow faster at optimal temperature and have a broader growth temperature range (27, 28). These complex differences in biological properties may reflect broad genetic variations between type A chromosomal *cpe* isolates and other *C. perfringens* strains. In fact, a newly identified product (Ssp4) of the *ssp4* gene (one of four *C. perfringens* genes encoding a small acid-soluble protein), was recently reported to be the most important protein for heat and nitrite resistance of spores made by chromosomal *cpe* strains (31). It was found that type A chromosomal *cpe* isolates producing resistant spores have a single amino acid substitution in their Ssp4 protein that mediates, in large part, their resistance phenotype (31).

Interestingly, most, if not all, of type A chromosomal *cpe* strains do not carry the θ toxin gene (*pfoA*), which indicates that chromosomal *cpe* strains produce non-hemolytic colonies on a sheep blood agar plate (15). This property is common, but not specific to chromosomal *cpe* strains, because some strains of type A plasmid *cpe*-positive and/or *cpe*-negative strains also do not carry the *pfoA* gene (15). In some cases, this property might help in the detection and identification of type A chromosomal *cpe C. perfringens* isolates.

## Conclusion

Recent advances in understanding the genetics of type A *cpe*-positive *C. perfringens* have facilitated the detection and identification of enterotoxigenic *C. perfringens* isolates in food-borne outbreaks and outbreaks of nosocomial diarrhea; using molecular assays to identify three types of the *cpe* loci in type A isolates, several novel insights into diseases caused by enterotoxigenic *C. perfringens* have been published; however, newly identified issues were also recognized in type A *cpe*-positive *C. perfringens* identification. In most isolates obtained from patient specimens, the current *cpe-*genotyping assays are useful tools; however, a small population of *cpe*-positive *C. perfringens*, including variant(s) of the *cpe* locus, has been found in the environment and these strains do not react appropriately in the current *cpe* genotyping assays (29, 43). The organization of the *cpe* locus in *C. perfringens* type A, C, D, and E has various arrangements (4, 32, 43); therefore, based on the sequence differences of the *cpe* locus of all types, molecular assays, including the *cpe*-genotyping assays for type A strains, should distinguish and identify these other *cpe* locus-carrying isolates. When developed, applying these assays to clinical isolates, human and veterinary GI diseases caused by enterotoxigenic *C. perfringens* should provide accurate and rapid diagnoses, allowing improved appreciation for the clinical significance of these strains.

## References

[1] Abrahao C, Carman RJ, Hahn H, Liesenfeld O (2001). Similar frequency of detection of *Clostridium perfringens* enterotoxin and *Clostridium difficile* toxins in patients with antibiotic-associated diarrhea. Eur J Clin Microbiol Infect Dis.

[2] Asha NJ, Wilcox MH (2002). Laboratory diagnosis of *Clostridium perfringens* antibiotic-associated diarrhea. J Med Microbiol.

[3] Augustynowicz E, Gzyl A, Slusarczyk J (2002). Detection of enterotoxigenic *Clostridium perfringens* with a duplex PCR. J Med Microbiol.

[4] Billington SJ, Wieckowski EU, Sarker MR, Bueschel D, Songer JG, McClane BA (1998). *Clostridium perfringens* type E animal enteritis isolates with highly conserved, silent enterotoxin sequences. Infect Immun.

[5] Borriello SP, Barclay FE, Welch AR, Stringer MF, Watson GN, Williams RK, Seal DV, Sullens K (1985). Epidemiology of diarrhea caused by enterotoxigenic *Clostridium perfringens*. J Med Microbiol.

[6] Borriello SP, Larson HE, Welch AR, Barclay F, Stringer MF, Bartholomew BA (1984). Enterotoxigenic *Clostridium perfringens*: a possible cause of antibiotic-assocaited diarrhea. Lancet.

[7] Brynestad S, Synstad B, Granum PE (1997). The *Clostridium perfringens* enterotoxin gene is on a transposable element in type A human food poisoning strains. Microbiology.

[8] Carman RJ, Sayeed S, Li J, Genheimer CW, Hiltonsmith MF, Wilkins TD, McClane BA (2008). *Clostridium perfringens* toxin genotypes in the feces of healthy North Americans. Anaerobe.

[9] Chalmers G, Bruce HL, Hunter DB, Parreira VR, Kulkarni RR, Jiang YF, Prescott JF, Boerlin P (2008). Multilocus sequence typing analysis of *Clostridium perfringens* isolates from necrotic enteritis outbreaks in broiler chicken populations. J Clin Microbiol.

[10] Collie RE, Kokai-Kun JF, McClane BA (1998). Phenotypic characterization of enterotoxigenic *Clostridium perfringens* isolates from non-foofborne human gastrointestinal diseases. Anaerobe.

[11] Collie RE, McClane BA (1998). Evidence that the enterotoxin gene can be episomal in *Clostridium perfringens* isolates associated with nonfoodborne human gastrointestinal diseases. J Clin Microbiol.

[12] Cornillot E, Saint-Joanis B, Daube G, Katayama S, Granum PE, Canard B, Cole ST (1995). The enterotoxin gene (*cpe*) of *Clostridium perfringens* can be chromosomal or plasmid-borne. Mol Microbiol.

[13] Czeczulin JR, Hanna PC, McClane BA (1993). Cloning, nucleotide sequencing, and expression of the *Clostridium perfringens* enterotoxin gene in *Escherichia coli*. Infect Immun.

[14] Daube G, Simon P, Limbourg B, Manteca C, Mainil J, Kaeckenbeeck A (1996). Hybridization of 2,659 *Clostridium perfringens* isolates with gene probes for seven toxins (α, β, ε, ι, theta, μ and enterotoxin) and for sialidase. Am J Vet Res.

[15] Deguchi A, Miyamoto K, Kuwahara T, Miki Y, Kaneko I, Li J, McClane BA, Akimoto S (2009). Genetic characterization of type A enterotoxigenic *Clostridium perfringens* strains. PLoS One.

[16] Fisher DJ, Fernandez-Miyakawa ME, Sayeed S, Poon R, Adams V, Rood JI, Uzal FA, McClane BA (2006). Dissecting the contributions of *Clostridium perfringens* type C toxins to lethality in the mouse intravenous injection model. Infect Immun.

[17] Garmony HS, Chanter N, French NP, Bueschel D, Songer JG, Titball RW (2000). Occurrence of *Clostridum perfringens* b2-toxin amongst animals, determined using genotyping and subtyping PCR assays. Epidemiol Infect.

[18] Harry KH, Zhou R, Kroos L, Melville SB (2009). Sporulation and enterotoxin (CPE) synthesis are controlled by the sporulation-specific sigma factors SigE and SigK in *Clostridium perfringens*. J Bacteriol.

[19] Heikinheimo A, Lindström M, Granum PE, Korkeala H (2006). Humans as reservoir for enterotoxin gene-carrying *Clostridium perfringens* type A. Emerg Infect Dis.

[20] Jost BH, Trinh HT, Songer JG (2006). Clonal relationship among *Clostridium perfringens* of porcine origin as determined by multilocus sequence typing. Vet Microbiol.

[21] Kaneko I, Miyamoto K, Mimura K, Yumine N, Utsunomiya H, Akimoto S, McClane BA (2011). Detection of enterotoxigenic *C. perfringens* in meat samples by using molecular methods. Appl Environ Microbiol.

[22] Kato H, Kato N, Watanabe K, Ueno K, Ushijima H, Hashira S, Abe T (1994). Application of typing by pulsed-field gel electrophoresis to the study of *Clostridium difficle* in a neonatal intensive care unit. J Clin Microbiol.

[23] Kobayashi S, Wada A, Shibasaki S (2008). Spread of a large plasmid carrying the *cpe* gene and the *tcp* locus amongst *Clostridium perfringens* isolates from nosocomial outbreaks and sporadic cases of gastrointestiritis in a geriatric hospital. Epidemiol Infect.

[24] Kokai-Kun JF, Songer JG, Czeczulin JR, Chen F, McClane BA (1994). Comparison of Western Immunoblots and gene detection assays for identification of potentially enterotoxigenic isolates of *Clostridium perfringens*. J Clin Microbiol.

[25] Lahti P, Heikinheimo A, Johansson T, Korkeala H (2008). *Clostridium perfringens* type A isolates carrying the plasmid-borne enterotoxin gene (genotype IS*1151*-*cpe* or IS*1470*-like-*cpe*) are a common cause of food poisoning. J Clin Microbiol.

[26] Lawrence G, Walker PD (1976). Pathogenesis of enteritis necroticans in Papua, New Guinea. Lancet.

[27] Li J, McClane BA (2006). Comparative effects of osmoptic, sodium nitrate-induced, and pH-induced stress on growth and survival of *Clostridium perfringens* type A isolates carrying chromosomal or plasmid-borne enterotoxin genes. Appl Environ Microbiol.

[28] Li J, McClane BA (2006). Further comparison of temperature effects on growth and survival of *Clostridium perfringens* type A isolates carrying a chromosomal or plasmid-borne enterotoxin gene. Appl Environ Microbiol.

[29] Li J, Sayeed S, McClane BA (2007). Prevalence of enterotoxigenic *Clostridium perfringens* isolates in Pittsburgh (Pennsylvania) area soil and home kitchens. Appl Environ Microbiol.

[30] Li J, Miyamoto K, McClane BA (2007). Comparison of virulence plasmids among *Clostridium perfringens* type E isolates. Infect Immun.

[31] Li J, McClane BA (2008). A novel small acid soluble protein variant is important for spore resistance of most *Clostridium perfringens* food poisoning isolates. PLoS Pathog.

[32] Li J, Miyamoto K, Sayeed S, McClane BA (2010). Organization of the *cpe* locus in CPE-positive *Clostridium perfringens* type C and D isolates. PLoS One.

[33] Lin YT, Labbe R (2003). Enterotoxigenicity and genetic relatedness of *Clostridium perfringens* isolates from retail foods in the United States. Appl Environ Microbiol.

[34] Lindström M, Heikinheimo A, Lathi P, Korkeara H (2011). Novel insight into the epidemiology of *Clostridium perfringens* type A food poisoning. Food Microbiol.

[35] McClane BA, Druse P (2005). Clostridial enterotoxins. Handbook on Clostridia.

[36] McClane BA, Doyle MP, Beuchat LR, Montville TJ (2001). Clostridium perfringens. Food Microbiology: Fundamentals and Frontier.

[37] Meer RR, Songer JG (1997). Multiplex polymerase chain reaction assay for genotyping *Clostridium perfringens*. Am J Vet Res.

[38] Miki Y, Miyamoto K, Kaneko-Hirano I, Fujiuchi K, Akimoto S (2008). Prevalence and characterization of enterotoxin gene-carrying *Clostridium perfringens* isolates from retail meat products in Japan. Appl Environ Microbiol.

[39] Miwa N, Nishina T, Kubo S, Atsumi M, Honda H (1998). Amount of enterotoxigenic *Clostridium perfringens* in meat detected by nested PCR. Int J Food Microbiol.

[40] Miyamoto K, Chakrabarti G, Morino Y, McClane BA (2002). Organization of the plasmid *cpe* locus in *Clostridium perfringens* type A isolates. Infect Immun.

[41] Miyamoto K, Wen Q, McClane BA (2004). Multiplex PCR genotyping assay that distinguishes between isolates of *Clostridium perfringens* type A carrying a chromosomal enterotoxin gene (*cpe*) locus, a plasmid *cpe* locus with an IS*1470*-like sequence, or a plasmid *cpe* locus with an IS*1151* sequence. J Clin Microbiol.

[42] Miyamoto K, Fisher DJ, Li J, Sayeed S, Akimoto S, McClane BA (2006). Complete sequencing and diversity analysis of the enterotoxin-encoding plasmids in *Clostridium perfringens* type A non-food-borne human gastrointestinal disease isolates. J Bacteriol.

[43] Miyamoto K, Yumine N, Mimura K, Nagahama M, Li J, McClane BA, Akimoto S (2011). Identification of novel *Clostridium perfringens* type E strains that carry an iota toxin plasmid with a functional enterotoxin gene. PLoS One.

[44] Mueller-Spitz SR, Stewart LB, Klump JV, McLellan SL (2010). Freshwater suspended sediments and sewage are reservoirs for enterotoxin-positive *Clostridium perfringens*. Appl Environ Microbiol.

[45] Myers GS, Rasko DA, Cheung JK (2006). Skewed genomic variability in strains of the toxinogenic bacterial pathogen *Clostridium perfringens*. Genome Res.

[46] Pillai SD, Vega E, Santo Domingo JW, Sadowsky MJ (2007). Molecular detection and characterization tools. Microbial Source Tracking.

[47] Popoff MR, Stiles BG, Druse P (2005). *Clostridial* toxins vs. other bacterial toxins. Handbook on Clostridia.

[48] Rooney AP, Swezey JL, Friedman R, Hecht DW, Maddox CW (2006). Analysis of core housekeeping and virulence genes reveals cryptic lineages of *Clostridium perfringens* that are associated with distinct disease presentation. Genetics.

[49] Sarker MR, Shivers RP, Sparks SG, Juneja VK, McClane BA (2000). Comparative experiments to examine the effects of heating on vegetative cells and spores of *Clostridium perfringens* isolates carrying plasmid versus chromosomal enterotoxin genes. Appl Environ Microbiol.

[50] Sayeed S, Fernandez-Miyakawa ME, Fisher DJ, Adams V, Poon R, Rood JI, Uzal FA, McClane BA (2005). Epsilon-toxin is required for most *Clostridium perfringens* type D vegetative culture supernatants to cause lethality in the mouse intravenous injection model. Infect Immun.

[51] Songer JG, Meer RR (1996). Genotyping of *Clostridium perfringens* by polymerase chain reaction is a useful adjunct to diagnosis of clostridial enteric disease in animals. Anaerobe.

[52] Tanaka D, Isobe J, Hosorogi S (2003). An outbreak of food-borne gastroenteritis caused *Clostridium perfringens* carrying the *cpe* gene on a plasmid. Jpn J Infect Dis.

[53] Tanaka D, Kimata K, Shimizu M (2007). Genotyping of *Clostridium perfringens* isolates collected from food poisoning outbreaks and healthy individuals in Japan based on the *cpe* locus. Jpn J Infect Dis.

[54] Urwin R, Maiden M (2003). Multi-locus sequence typing: a tool for global epidemiology. Trends Microbiol.

[55] Van Damme-Jongsten M, Rodhouse J, Gilbert RJ, Notermans S (1990). Synthetic DNA probes for detection of enterotoxigenic *Clostridium perfringens* strains isolated from outbreaks of food poisoning. J Clin Microbiol.

[56] Watanabe M, Hitomi S, Sawahara T (2008). Nosocomial diarrhea caused by *Clostridium perfringens* in the Tsukuba-Tsuchiura district, Japan. J Infect Chemother.

[57] Wen Q, McClane BA (2004). Detection of enterotoxigenic *Clostridium perfringens* type A isolates in American retail foods. Appl Environ Microbiol.

[58] Wen Q, Miyamoto K, McClane BA (2003). Development of a duplex PCR genotyping assay for distinguishing *Clostridium perfringens* type A isolates carrying chromosomal enterotoxin (*cpe*) genes from those carrying plasmid-borne enterotoxin (*cpe*) genes. J Clin Microbiol.

[59] Zhao Y, Melville SB (1998). Identification and characterization of sporulation-dependent promoters upstream of the enterotoxin gene (*cpe*) of *Clostridium perfringens*. J Bacteriol.

